# “Distemper” in the Cat

**Published:** 1899-06

**Authors:** 


"DISTEMPER" IN THE CAT. The word *distemper* is here used simply by way of analogy, since I have on several occasions in private and in hospital work witnessed in this animal a condition which in certain symptoms, course, and other peculiarities resembles the disease designated by that name in the dog. There is no proof that establishes the identity of the two diseases, although canine distemper is communicable to some of the wild species of that genus.

The disease affects young cats usually from six to eight months old. They may or may not be associated with dogs. The period of incubation could not be determined.

As to symptoms, the cat becomes dull and mopish, impaired appetite, catarrhal condition of the respiratory mucous membrane, sneezing, discharge from the nose adhering to the nostrils, snuffled breathing, and sometimes cough; also conjunctivitis, with a watery discharge from the eyes. The stools are variable; sometimes soft and fetid.

I have not been able to detect any vesicular eruption in the abdominal skin.

The prognosis seems to be favorable. I have not seen the usual complications of canine distemper, nor any fatal cases.

The treatment is simple. Good hygiene, stimulant tonics, such as nux vomica and cinchona, laxatives when there is constipation, bismuth, laudanum, camphor, creolin or carbolic acid when there is fetid diarrhœa, and whiskey, milk, and eggs when the prostration and inappetence are marked.

